# SCUBA implements a storage format-agnostic API for single-cell data access in R

**DOI:** 10.12688/f1000research.154675.1

**Published:** 2024-10-21

**Authors:** William M. Showers, Jairav Desai, Krysta L. Engel, Clayton Smith, Craig T. Jordan, Austin E. Gillen

**Affiliations:** 1 Division of Hematology, University of Colorado Anschutz Medical Campus School of Medicine, Aurora, Colorado, USA; 2 RefinedScience, Aurora, Colorado, USA; 3 Rocky Mountain Regional VA Medical Center, Aurora, Colorado, USA

**Keywords:** single-cell sequencing, multimodal, software tools, R package, Python, visualization

## Abstract

While robust tools exist for the analysis of single-cell datasets in both Python and R, interoperability is limited, and analysis tools generally only accept one object class. Considerable programming expertise is required to integrate tools across package ecosystems into a comprehensive analysis, due to their differing languages and internal data structures. This complicates validation of results and leads to inconsistent visualizations between analysis suites. Conversion between object formats is the most common solution, but this is difficult and error-prone due to the rapid pace of development of the analysis suites and their underlying data structures. To address this, we created SCUBA (Single-Cell Unified Backend API), an R package that implements a unified data access API for all common R and Python single-cell object formats. SCUBA extends the data access approach from the widely used Seurat package to SingleCellExperiment and anndata objects. SCUBA also implements new data-specific access functions for all supported object types. Performance scales well across all SCUBA-supported formats. In addition to performance, SCUBA offers several advantages over object conversion for the visualization and further analysis of pre-processed single-cell data. First, SCUBA extracts only data required for the operation at hand, leaving the original object unmodified. This process is simpler, less error prone, and less memory intensive than object conversion, which operates on the entire dataset. Second, code written with SCUBA can use any supported object class as input, with simple and consistent syntax across object formats. This allows a single analysis script or package (like our interactive single-cell browser, scExploreR) to work seamlessly with multiple object types, reducing the complexity of the code and improving both readability and reproducibility. Adoption of SCUBA will ultimately improve collaboration and reproducible research in single-cell analysis by lowering the barriers between package ecosystems.

## Introduction

**Figure 1.**
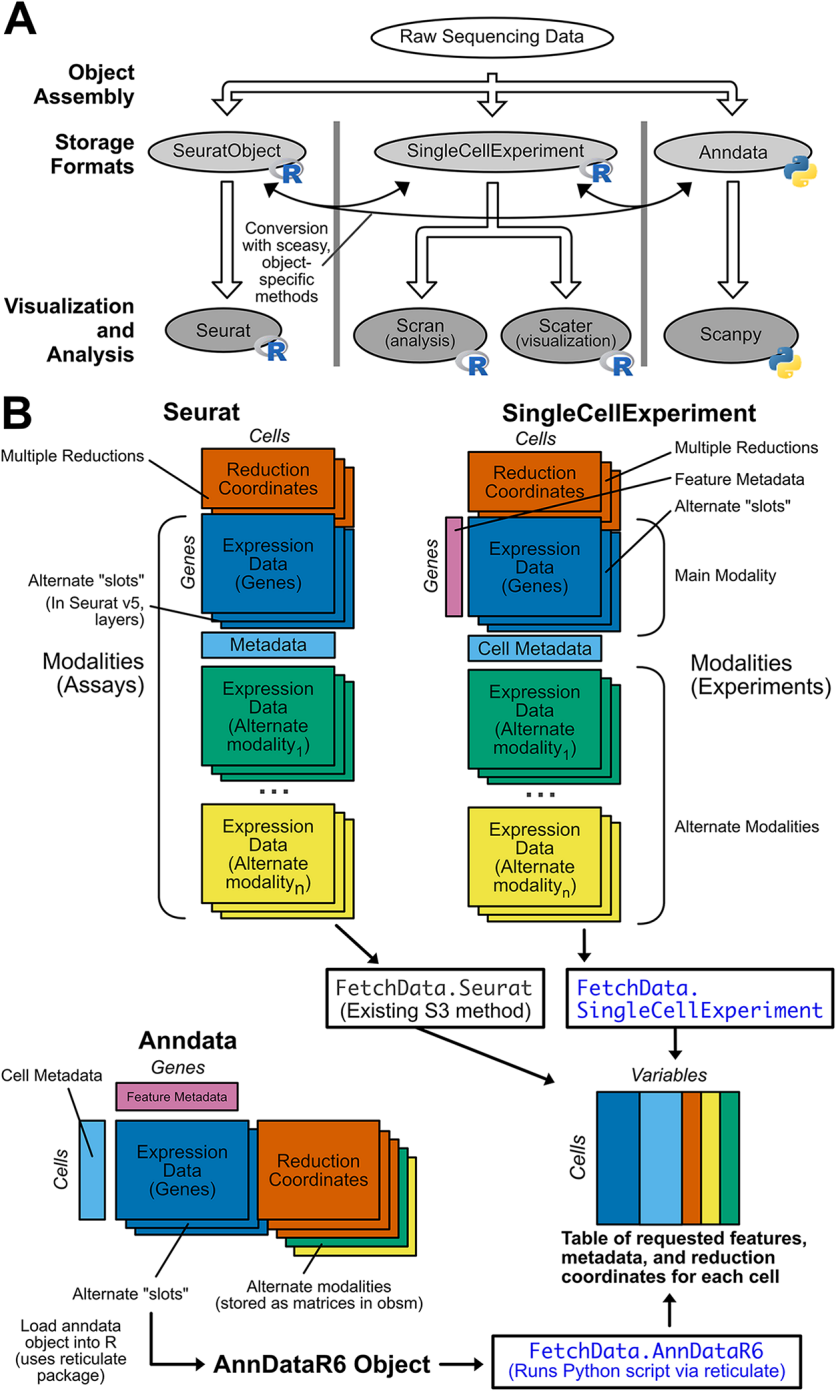
SCUBA addresses challenges posed by multiple object formats in single-cell sequencing data. A) Raw single-cell sequencing data is stored in defined object classes, and processed downstream by packages that only accept one object class. This creates “walled garden” analysis suites of incompatible packages that complicate single-cell analysis. When a specific downstream package is desired, the user will need to convert between object formats prior to use. This is possible, but the process is difficult and error-prone. B) SCUBA returns feature expression data, metadata, and reduction coordinates from Seurat, SingleCellExperiment, and Anndata objects in a consistent output format. An overview of each object structure is shown, with rectangles indicating data matrices stored in each object. Dimensions of the matrices are labeled with “cells” or “genes” (features), and matrices placed adjacent to one another indicate requirements that matrices have the same number of values in the dimension indicated (i.e. for Seurat objects, the “reduction coordinates” and “gene expression” matrices must have the same number of cells, but may have varying number of genes (or in the case of reduction coordinates, dimensions). Next to the description of each matrix, object-specific code to retrieve the matrix is given. If additional modalities are supported by an object type, the structure of matrices specific to modalities are shown, along with code to retrieve data on alternate modalities. The output format for SCUBA is shown at the bottom of the panel. The output is a single R data.frame with values for each variable requested for each cell. The S3 methods added by SCUBA to yield the output format are shown in blue, and are based on the existing FetchData generic and method from Seurat.

The rapidly evolving landscape of single-cell sequencing methods has led to the production of increasingly large and diverse single-cell datasets, greatly improving our knowledge of both inter- and intra-patient heterogeneity in a wide range of diseases and normal tissues. ^1, 2^ While there are many excellent tools available for analyzing single-cell datasets in both the Python and R ecosystems, interoperability is hindered by the use of incompatible object classes ( Figure 1A). Analysis tools generally only accept one object class, forcing users to commit to a suite of packages at the beginning of an analysis. This restriction limits access to tools outside that suite, creating “walled gardens” that pose several challenges for single-cell analysis. Single-cell analysis can be inaccessible to bench scientists due to the programming experience required, and different object formats make it even more difficult for biologists to analyze data. The implementation of popular object formats in both Python and R requires users to be fluent in both programming languages, and it is difficult and time consuming to learn both without formal education in data structures and syntax in each language. The widespread use of multiple object formats also makes it difficult to validate results produced by one analysis suite with another, and visualizations produced with different analysis suites are not consistent. Additionally, the practical analysis of objects with large numbers of cells requires a way to interact with on-disk matrices rather than loading all data in memory. On-disk matrix implementations are analysis suite-specific, which introduces additional barriers to effective analysis. For example, anndata objects are natively stored in the memory-efficient HDF5 format, but anndata objects are not compatible with the Bioconductor’s single-cell tools or Seurat. If a user converts an anndata object to Seurat or SingleCellExperiment format, they must use a different on-disk matrix implementation specific to that format, which further restricts the analyses that can be performed. If single-cell object formats were interoperable, it would be easy for researchers to analyze data from any single-cell dataset, regardless of the object format used when the dataset was generated, but unfortunately this is not the case.

Currently, the most effective solution is to manually convert between object classes. All major single-cell analysis packages implement conversion functions, and third-party packages such as sceasy ^3^ are specifically designed for these conversions. However, inconsistencies in approaches to object structure across implementations often result in data loss upon conversion, which is difficult to overcome. Additionally, the rapid development of these packages means that conversion functions are difficult to maintain and frequently break due to changes in other packages. This is especially true when converting to and from the anndata format, since this format is implemented in the Python programming language, and the Seurat and SingleCellExperiment objects are implemented in the R programming language. Even if conversion is successfully achieved without loss of data quality, it has recently been demonstrated that results from Seurat differ from those of Scanpy, ^4^ despite the fact the two packages implement ostensibly identical processing steps. Addressing interoperability and consistency issues between analysis suites is crucial to ensuring the fidelity and reproducibility of single-cell analysis results, making consistent visualizations across suites essential.

Rather than conversion between objects, we propose a more sustainable approach to the visualization and further analysis of pre-processed single-cell data by implementing a unified API for all common single-cell object formats. Here, we present Single-Cell Unified Backend API (SCUBA), an R package based on the data accession function in the widely used Seurat ^5^ package that returns data from Seurat, SingleCellExperiment, and annadata objects in a common format for downstream visualization and analysis ( Figure 1B). SCUBA also implements new data-specific access functions for all supported object types. Data is returned in a single R data.frame, with requested variables as columns, and cells as rows. The functions in this package allow users to plot data in a consistent manner from these object types in R, without requiring conversion. SCUBA can also be used in functional programming applications as the basis for single-cell plotting packages, or in the development of Shiny apps. For objects with very large numbers of cells, it is now possible to choose the object class based on on-disk storage performance and produce visually consistent plots without having to downsample the object. Packages and scripts created with SCUBA are flexible with regard to input type, greatly improving the consistency of results between objects and increasing accessibility of these analyses for non-programmers.

## Methods

### Implementation

SCUBA provides a unified framework for data access by leveraging R’s S3 object-oriented programming. ^6^ The workflow for data access in SCUBA is based on Seurat’s FetchData method. We implemented a new generic that uses the Seurat FetchData method for Seurat objects, and novel methods for SingleCellExperiment and anndata objects. The generic was chosen as a basis due to its ease of use, and its implementation in Seurat’s plotting functions, which are widely used.

S3 methods were created in SCUBA to extend the existing FetchData generic to SingleCellExperiment and anndata objects. Access to the anndata objects is accomplished using reticulate ^7^ and performing as many operations in python as possible before returning data to R. The workflow from the existing method for Seurat objects is largely unchanged upon re-implementation for these object formats. We used code from the Seurat package under the terms of the package’s MIT license.

In addition to replicating the behavior of FetchData in SingleCellExperiment and anndata objects, SCUBA includes S3 generics and methods specific to the retrieval of metadata and reduction coordinates from each object format. These methods offer improvements in performance relative to retrieving the same data via FetchData for large objects.

### Operation

SCUBA can be installed as an R package via GitHub using the devtools ^8^ R package, and can be used on all common operating systems. To ensure compatibility across operating systems, SCUBA is maintained using Continuous Integration (CI), with Github Actions and the testthat ^9^ R package. The Github Actions workflow performs 100+ tests on recent Linux R and Python versions whenever a pull request is created. Tests are additionally run on Mac OS and Windows for releases. The dataset used for testing is a downsampled version of the acute myeloid leukemia reference dataset ^10^ from Triana et al. 2021. ^11^

If using SCUBA with Seurat or SingleCellExperiment objects, no further installation is necessary beyond the R dependencies. For anndata objects, the reticulate ^7^ R package and a Python installation are required. The following python packages must be manually installed: pandas, ^12^ numpy, ^13^ scipy, ^14^ and anndata. ^15^ We recommend installing these packages in an anaconda ^16^ environment and loading the environment in R with reticulate::use_condaenv(), but this is not required. Detailed installation instructions are available on the SCUBA GitHub Page.

## Use cases

The features of SCUBA fall broadly into three categories; data access, data visualization, and data exploration. The functions provided in these categories can be used independently or in a stepwise pipeline. Generally speaking, SCUBA works best for objects that have been filtered and clustered, though SCUBA can work on objects in any state as long as the data being requested exists. Here we highlight independent use cases using a downsampled version of the acute myeloid leukemia reference dataset ^10^ generated by Triana et al. ^11^ Additional vignettes are provided on the SCUBA GitHub page.

### FetchData Methods for SingleCellExperiment and Anndata Objects

**Figure 2.**
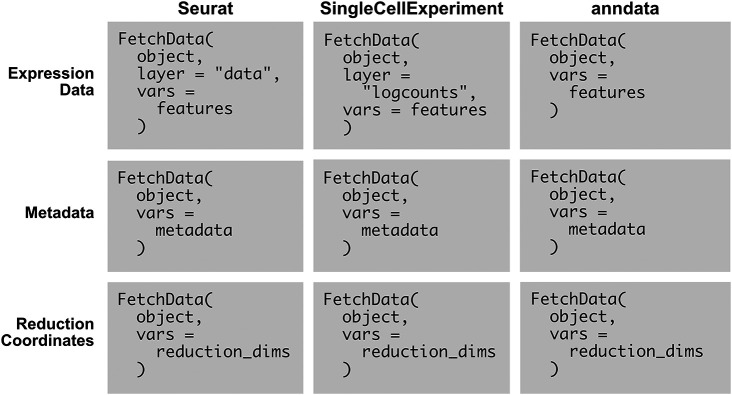
The methods added by SCUBA simplify the retrieval of data from supported object classes. The existing seurat method (first column), is compared to the methods added by SCUBA for SingleCellExperiment and anndata objects (second and third columns). The methods use consistent syntax across object classes, and involve the use of only a few parameters. Pseudocode is used in the examples. object represents a single-cell object. features represents one or more features, from any modality in the object. metadata represents one or more metadata variables, for example, cell type classifications. reduction_dims represents a set of dimensions in a reduction included with the object, with the number of the dimension separated from the reduction with an underscore. For example, to fetch the first and second dimensions of the UMAP projection, reduction_dims would be c(“UMAP_1”, “UMAP_2”).

Example usage of SCUBA’s FetchData methods is given Figure 2. The existing Seurat method (first column) is compared to the methods added by SCUBA. There are only minor variations in input syntax across the three supported object types, and the required parameters are few, making the methods easy to use. All data requested is specified using the vars parameter. The methods infer whether the data requested is metadata, reduction coordinates, or feature expression by parsing the character vector passed to this parameter. To retrieve feature expression or reduction coordinates, the user adds a “key” with an underscore giving the name of the reduction, or the modality to pull feature expression data from. If using an object with only one modality, the modality key is not needed, and the key is also not needed to retrieve metadata. Minor variations in the key exist between object types, due to object-specific conventions for naming modalities (which are called “assays” in Seurat objects, and “Experiments” in SingleCellExperiment objects). Variations in the layer parameter are based on variations in conventions for naming layers (in SingleCellExperiment objects, “assays”, and in Seurat v4 and earlier, “slots”). The consistency in parameters between the three object types, and the presence of only minor differences in inputs to each parameter, facilitates the writing of scripts for any object type.

The output of FetchData is identical across the three object classes. The output is an R data.frame with values for each requested feature in vars per cell. Columns represent each feature, and rows represent cells.

### Metadata, reduction-specific accession methods

**Figure 3.**
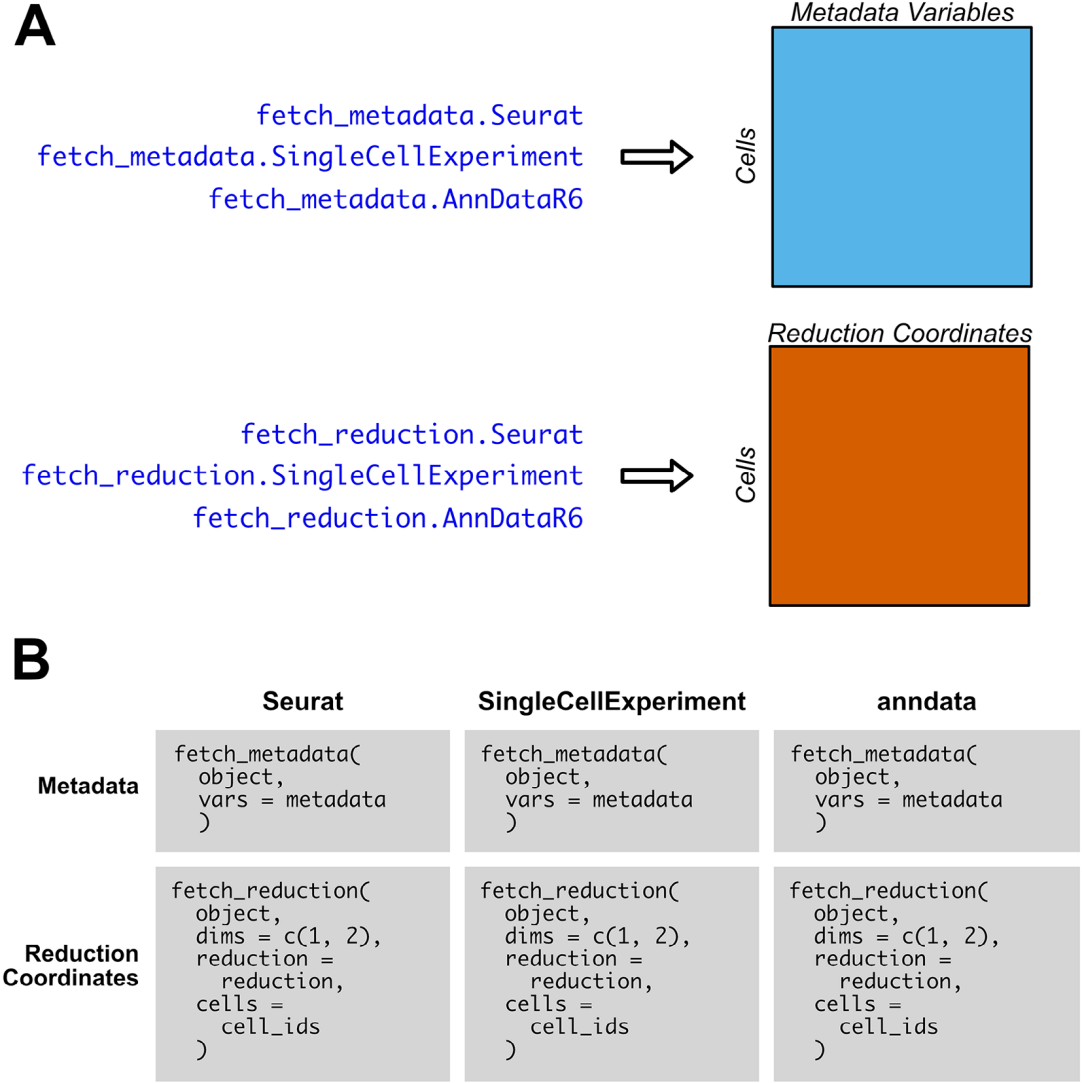
SCUBA methods specific to the retrieval of metadata and reduction coordinates. A) Overview of outputs of fetch_metadata, for metadata variables, and fetch_reduction, for reduction coordinates. The output is an R data.frame with the metadata or reduction coordinates as columns, and the cells as rows. B) Comparison of the usage of fetch_metadata and fetch_reduction across each object type. For fetch_metadata, the metadata variable or variables to retrieve (which are represented as metadata in this pseudocode example) are specified via a character vector input to vars. For fetch_reduction, the dimensions to return from the reduction coordinate matrix is passed to dims, and the reduction to pull from is specified via reduction. The cells parameter allows the user to specify which cells to fetch reduction coordinates for. For ease of use and flexibility, there is no difference in inputs between object types; only the object itself varies.

SCUBA also includes S3 generics and methods specific to the retrieval of metadata and reduction coordinates, which are faster than retrieving the same data via FetchData for R object types. An overview of the fetch_metadata and fetch_reduction functions is given in Figure 3A. As with FetchData, the output of fetch_metadata and fetch_reduction is an R data.frame with data for the requested metadata variables or reduction coordinates, respectively, as columns, and rows for each cell. Figure 3B compares the usage of fetch_reduction and fetch_metadata between supported object types. We implement these methods in anndata objects for consistency in syntax, but their performance is roughly equivalent to FetchData. The functions are easy to use, and the inputs to each function do not vary based on object type. To set defaults for the reduction and cells parameters of fetch_reduction, SCUBA provides several utility methods. default_reduction will search for UMAP, t-SNE, and PCA reductions, and will return them in that order if they exist. get_all_cells will return the IDs of all cells in the object.

**Figure 4.**
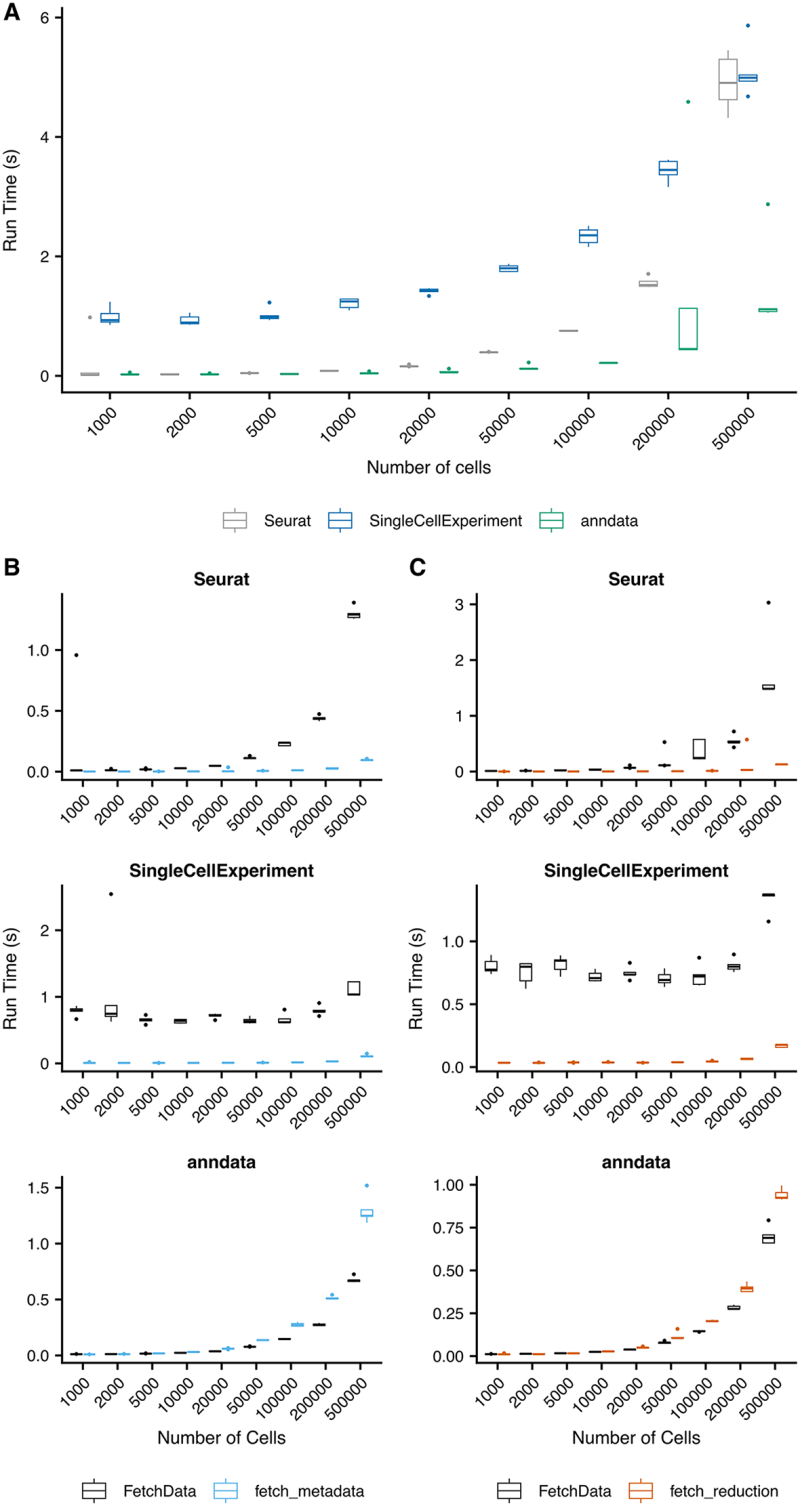
Performance testing of SCUBA functions and methods. Five random subsets of the indicated numbers of cells were created from the Human Brain Atlas object downloaded from CellXGene. ^21^ The subsets were saved in the following object formats: Seurat, via saveRDS(), SingleCellExperiment, via HDF5Array::saveHDF5SummarizedExperiment(), and anndata, via write_h5ad. For all tests, the indicated operations were run on each of the five subsets, and the run time was measured using the tictoc ^22^ package. A) Comparison of FetchData methods vs. fetch_metadata for the retrieval of data for a single metadata variable. In most cases, using fetch_metadata to pull metadata was more performant than using FetchData. B) Comparison of FetchData methods vs. fetch_reduction for the retrieval of data for a pair of reduction coordinates. fetch_reduction was more performant than FetchData for the retrieval of reduction coordinates in most cases. C) Performance of the FetchData methods developed for SingleCellExperiment and anndata objects, compared to the existing FetchData method for Seurat objects. A single feature was pulled via FetchData for each of the random subsets for the indicated object and number of cells.

Figure 4A-B compares the performance of fetch_metadata and fetch_reduction with the performance of FetchData to pull one metadata variable, and the first and second dimensions of UMAP coordinates, respectively. Run time was tested for each function on random subsets of varying numbers of cells, with five subsets created for each size. The fetch_metadata and fetch_reduction methods were more performant than FetchData in Seurat and SingleCellExperiment objects for all subsets tested. In anndata objects, the runtime of these functions was comparable to that of FetchData. Performance testing for the FetchData methods added by SCUBA was also performed ( Figure 4C). Performance of the method for anndata objects exceeds the performance for the existing Seurat method in most cases, and the performance of the SingleCellExperiement method exceeds performance of the Seurat method for the largest subset tested (500k cells).

### Example scripts created with SCUBA

**Figure 5.**
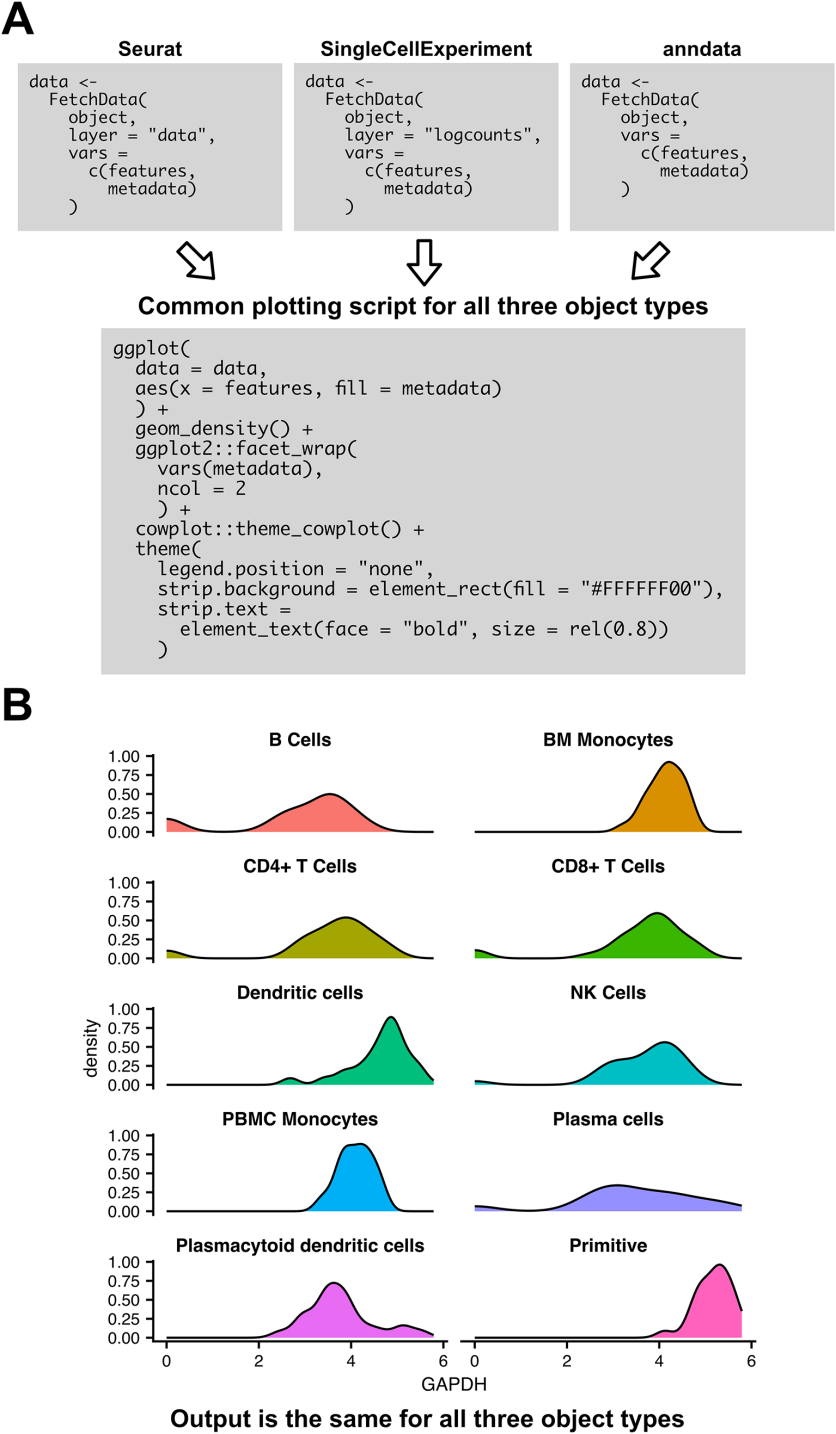
SCUBA enables flexible plotting scripts harmonized across object types. A) Example script for visualizing expression of a gene by cluster in a density plot for each of the three supported object types. The three boxes for FetchData indicate slight variations in the script for each object type. All downstream code is the same across object formats. B) Output of the plotting script in (A). Output does not vary by object type.

Figure 5 gives an example usage of SCUBA methods to create plots with consistent visuals across object types. Figure 5A shows the scripts to create a density plot showing expression by cell type from each of the three supported object types, showing regions of the script that vary between object types, and regions that are conserved. Figure 5B shows the output of the example script. The script demonstrates the ease at which expression data can be visualized from each object format, and the ease at which plot visuals can be harmonized across object formats.

Any plot visualizing a combination expression data, metadata, and reduction coordinates can be created by generating a table from FetchData, fetch_metadata, or fetch_reduction, and passing the output table to downstream plotting code. Plotting is performed via ggplot2 ^17^ in this example, but any other plotting package that accepts a data.frame or a tibble as input may be used. If desired, it is also possible to convert to a pandas ^12^ dataframe via Reticulate, ^7^ and perform plotting operations in python. The flexibility of SCUBA’s data access methods facilitate the creation of a broad variety of plots from single-cell data.

**Figure 6.**
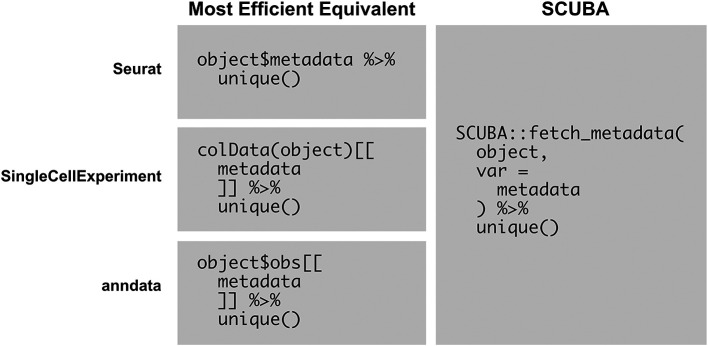
SCUBA simplifies common object exploration operations. This figure compares the usage of SCUBA with the most efficient equivalent operations for viewing the unique values of a metadata variable represented in an object. The operation with SCUBA is shown in the first column, and the most efficient equivalents are shown in the second column. SCUBA simplifies this operation, allowing for the development of scripts that are generalized for multiple object types.

Figure 6 shows an example script that simplifies the printing of unique values of a metadata variable represented in an object, which is a commonly used basic operation in analysis. With SCUBA, this operation can simply be performed by calling fetch_metadata on the object and piping the results to unique(). The language used is the same for all supported object classes, which negates the need to memorize and use the most efficient function calls for each respective object type.

## Conclusions

SCUBA addresses issues with interoperability between single-cell object formats by providing a flexible backend that returns data in a consistent format, via a consistent interface. The consistent output format of SCUBA facilitates downstream use in functional programming applications (plotting scripts, packages, etc.) and allows for consistent visualizations across object types. Packages and scripts using SCUBA will not require object conversion prior to use, conferring several advantages for end users. Users will not have to risk data loss upon object conversion, and analysis will be more straightforward without conversion, requiring less programming experience. Packages made with SCUBA will also allow users to choose object classes based on storage and performance characteristics that are best for the specific dataset, rather than being constrained to a class based on downstream packages. SCUBA does not allow users to use any analysis package with any object format, however. The aforementioned benefits only apply to packages and scripts created using SCUBA. SCUBA also only performs data access operations, and is not for object assembly, clustering, or filtering. Because of this, SCUBA is not a replacement for analysis packages such as Seurat and Scanpy. Instead, SCUBA allows users to visualize objects that have been prepared with these analysis packages in the same manner, regardless of object class.

Support for MuData ^18, 19^ will be added in the future, as this Python object class is especially useful for storing data from multimodal single-cell sequencing experiments. SCUBA is particularly well suited for interactive use, such as in Shiny apps, where multiple object formats may be used as inputs. We developed a single-cell browser, scExploreR, ^20^ that allows users to create consistent Seurat-style visualizations from either Seurat, SingleCellExperiment, or anndata objects. SCUBA can also be used to create a plotting package that produces visuals from any supported object class for reports and shiny apps, and a QC package reporting the results of preprocessing steps such as filtering, clustering, and batch correction could also be created using SCUBA. The flexibility of SCUBA is envisioned to facilitate analysis and visualization of preprocessed data, unifying disparate object-based package ecosystems.

## Ethics and consent

Ethical approval and consent were not required.

## Data Availability

SCUBA uses two third-party datasets for performance benchmarking, testing, and demonstration in the manuscript. The datasets are described below. Figshare: Expression of 197 surface markers and 462 mRNAs in 15281 cells from blood and bone marrow from a young healthy donor. https://doi.org/10.6084/m9.figshare.13398065.v4. ^10^ This project contains the following underlying data:
• 200AB_projected.rds. (Seurat object with 15821 cells, showing the expression of 197 surface markers and 462 mRNAs in bone marrow from a young healthy donor). The dataset is available under the terms of the Creative Commons Attribution 4.0 International license (CC-BY 4.0). CELLxGENE: Human Brain Cell Atlas v1.0. https://cellxgene.cziscience.com/collections/283d65eb-dd53-496d-adb7-7570c7caa443. This project contains the following underlying data:
• cc9bfb86-96ed-4ecd-bcc9-464120fc8628.rds. (Seurat object with 800k non-neuronal cells used for performance benchmarking in the manuscript. The file is accessed by selecting “All non-neuronal cells” and then the.rds radio button). The dataset is available under the terms of the Creative Commons Attribution 4.0 International license (CC-BY 4.0). The Velten et al. dataset ^10^ was processed to yield a format suitable for testing and demonstration of SCUBA, downsampled, and stored in the inst/extdata/ and data/directories of the SCUBA repo. Scripts used in these operations and performance benchmarking are available at the manuscript GitHub repo: https://github.com/amc-heme/SCUBA_Manuscript. Working examples of code shown in figures 2, 3, 5, and 6 are also stored in this repo. Software, up to date source code, and tutorials are available from: https://github.com/amc-heme/scuba Archived source code at time of publication: https://zenodo.org/doi/10.5281/zenodo.13776167 License: MIT

## References

[1] Schäfer PSL Dimitrov D Villablanca EJ : Integrating single-cell multi-omics and prior biological knowledge for a functional characterization of the immune system. *Nat. Immunol.* 2024;25:405–417. 10.1038/s41590-024-01768-2 38413722

[2] Zeng AGX : A cellular hierarchy framework for understanding heterogeneity and predicting drug response in acute myeloid leukemia. *Nat. Med.* 2022;28:1212–1223. 10.1038/s41591-022-01819-x 35618837

[3] Kiselev V Huang N : sceasy. 2022.

[4] Wolf FA Angerer P Theis FJ : SCANPY: large-scale single-cell gene expression data analysis. *Genome Biol.* 2018;19:15. 10.1186/s13059-017-1382-0 29409532 PMC5802054

[5] Hao Y : Dictionary learning for integrative, multimodal and scalable single-cell analysis. *Nat. Biotechnol.* 2024;42:293–304. 10.1038/s41587-023-01767-y 37231261 PMC10928517

[6] Wickham H : S3. *Advanced R.* Chapman and Hall/CRC;2019. 10.1201/9781351201315-16

[7] Ushey K Allaire J Tang Y : reticulate: Interface to ‘Python’. 2023.

[8] Wickham H Hester J Chang W : devtools: Tools to Make Developing R Packages Easier. 2022.

[9] Wickham H : testthat: Get Started with Testing. *The R Journal.* 2011;3:5. 10.32614/RJ-2011-002

[10] Velten L Triana S Haas S : Expression of 197 surface markers and 462 mRNAs in 15281 cells from blood and bone marrow from a young healthy donor.[Dataset]. *Figshare.* 2021. 10.6084/m9.figshare.13398065.v4

[11] Triana S : Single-cell proteo-genomic reference maps of the hematopoietic system enable the purification and massive profiling of precisely defined cell states. *Nat. Immunol.* 2021;22:1577–1589. 10.1038/s41590-021-01059-0 34811546 PMC8642243

[12] The pandas development team: Pandas. 2023. 10.5281/ZENODO.3509134

[13] Harris CR : Array programming with NumPy. *Nature.* 2020;585:357–362. 10.1038/s41586-020-2649-2 32939066 PMC7759461

[14] Virtanen P : Author Correction: SciPy 1.0: fundamental algorithms for scientific computing in Python (Nature Methods, (2020), 10.1038/s41592-019-0686-2). *Nat. Methods.* 2020;17:352–352. 10.1038/s41592-020-0772-5 32094914 PMC7056641

[15] Virshup I Rybakov S Theis FJ : anndata: Annotated data. 2021. 2021.12.16.473007. 10.1101/2021.12.16.473007

[16] Conda contributors: Conda: A system-level, binary package and environment manager running on all major operating systems and platforms. 2024.

[17] Wickham H : *Ggplot2: Elegant Graphics for Data Analysis.* Switzerland: Springer;2016. 10.1007/978-3-319-24277-4

[18] Bredikhin D Kats I Stegle O : MUON: multimodal omics analysis framework. *Genome Biol.* 2022;23:42. 10.1186/s13059-021-02577-8 35105358 PMC8805324

[19] Virshup I : The scverse project provides a computational ecosystem for single-cell omics data analysis. *Nat. Biotechnol.* 2023;41:604–606. 10.1038/s41587-023-01733-8 37037904

[20] Showers W Desai J Gipson S : scExploreR: a Flexible Shiny App for Democratized Analysis of Multimodal single-cell RNA-seq Data. 2024.

[21] Siletti K : Human Brain Cell Atlas v1.0.[Dataset]. *CELLxGENE.* Reference Source 2023.

[22] Izrailev S : tictoc: Functions for Timing R Scripts, as Well as Implementations of ‘Stack’ and ‘StackList’ Structures. 2023.

