# Supplementary material for: SCUBA implements a storage format-agnostic API for single-cell data access in R
Source: F1000Res. 2025 Jun 2;13:1256. Originally published 2024 Oct 21. [Version 2] doi: 10.12688/f1000research.154675.2 (PMC12351237; doi:10.12688/f1000research.154675.2)
Supplement: Peer review history

# Reviewer report: Reviewer response for version 2

**DOI:** 10.5256/f1000research.182803.r396950

**Published:** 2025-08-19

**Authors:** Benedikt Obermayer

**Affiliations:** 1 Berlin Institute of Health at Charité – Universitätsmedizin Berlin, Berlin, Germany

This manuscript presents an R package for unified data access to single-cell genomics objects from different commonly used formats (Seurat, SingleCellExperiment, and anndata). In version 2, most issues raised by previous reviewers were satisfactorily addressed, and the paper has reached a sufficiently sound stage.

 I have one more major issue and a few small corrections that could be incorporated.

 Major issue:

 I agree with Damian Panas and Marcin Tabaka in that this package does not really present a significant advancement or novel solution to comprehensively address the problem of interoperability. People who manage to successfully set up reticulate and conda environments to be used within their R installation would be expected to be able to get necessary data out of objects in different formats. People with less expertise for which a visualization / data exploration tool allowing for different input formats might be most useful will probably not be able to set this up in a reasonable time frame. In that case, I'd prefer a web-based or maybe Docker-based explorer that accepts R as well as python objects as input.

 Minor issues:

 - I don't really understand the advantage of SCUBA over the anndata R package for reading h5ad files and accessing their contents, apart from a unified syntax. (However, anndata specific data structures such as the ad$raw slot don't seem to be accessible using SCUBA). SCUBA appears to be somewhat faster than anndata in my hands, but that is probably of little concern to most users. This could be explained better.

 - I noticed two typos (Suerat instead of Seurat in Fig. 1 caption, and SueratWrapper instead of SeuratWrapper on p4 bottom).

 - When installing the package, my existing conda env with anndata did not have a sufficiently recent version (including anndata.abc, which I think was introduced in 0.10). Requirements don't specify this

 - the plot functions still use the deprecated FetchData method. Why is density plot not part of the package if it's used in the User Guide?

Are the conclusions about the tool and its performance adequately supported by the findings presented in the article?

Partly

Is the rationale for developing the new software tool clearly explained?

Yes

Is the description of the software tool technically sound?

Yes

Are sufficient details of the code, methods and analysis (if applicable) provided to allow replication of the software development and its use by others?

Yes

Is sufficient information provided to allow interpretation of the expected output datasets and any results generated using the tool?

Yes

Reviewer Expertise:

Bioinformatics, single-cell genomics, computational biology

I confirm that I have read this submission and believe that I have an appropriate level of expertise to confirm that it is of an acceptable scientific standard.

# Reviewer report: Reviewer response for version 2

**DOI:** 10.5256/f1000research.182803.r389307

**Published:** 2025-08-13

**Authors:** Marcin Tabaka, Damian Panas

**Affiliations:** 1 International Centre for Translaional Eye Research, Institute of Physical Chemistry, Polish Academy of Sciences, Warsaw, Poland; 2 International Centre for Translational Eye Research, Institute of Physical Chemistry, Polish Academy of Sciences, Warsaw, Mazowieckie, Poland; 3 ICTER, Institute of Physical Chemistry, Polish Academy of Sciences, Warsaw, Poland

The revised article comprises significant improvements on the initial submission. The comprehensive documentation, created using the pkgdown framework, is an essential addition. The documentation is now clearly structured, detailed, and supplemented by numerous examples. The authors have reformatted the manuscript to improve clarity and expanded the benchmarking section to provide a more informative performance comparison. Another significant addition is the new fetch_data() function, which resolves one of the most important issues raised in the initial submission. While a few minor problems presented earlier remain, the most critical concerns have been addressed and resolved. Overall, the current version of the SCUBA is a robust, high-quality tool that demonstrates thoughtful improvements in its usability.

Are the conclusions about the tool and its performance adequately supported by the findings presented in the article?

No

Is the rationale for developing the new software tool clearly explained?

Partly

Is the description of the software tool technically sound?

Partly

Are sufficient details of the code, methods and analysis (if applicable) provided to allow replication of the software development and its use by others?

Partly

Is sufficient information provided to allow interpretation of the expected output datasets and any results generated using the tool?

Yes

Reviewer Expertise:

Single-cell Genomics, Bioinformatics

We confirm that we have read this submission and believe that we have an appropriate level of expertise to confirm that it is of an acceptable scientific standard.

# Reviewer report: Reviewer response for version 1

**DOI:** 10.5256/f1000research.169727.r350233

**Published:** 2025-01-29

**Authors:** Marcin Tabaka, Damian Panas

**Affiliations:** 1 International Centre for Translational Eye Research, Institute of Physical Chemistry, Polish Academy of Sciences, Warsaw, Mazowieckie, Poland; 2 ICTER, Institute of Physical Chemistry, Polish Academy of Sciences, Warsaw, Poland; 3 International Centre for Translaional Eye Research, Institute of Physical Chemistry, Polish Academy of Sciences, Warsaw, Poland

In this study, William M. Showers and colleagues developed SCUBA, an R package designed to facilitate the access to single-cell data stored in various formats commonly utilized by single-cell data analysis software in R (Seurat, Scran) or Python (Scanpy). These single-cell data storage formats include SeuratObject (Seurat), SingleCellExperiment (Scran, Bioconductor’s single-cell tools), Anndata (Scanpy). Interoperability between R-based and Python-based tools and data types is a real problem in bioinformatics. Several tools are typically used in this case, such as zellkonverter, anndataR, sceasy, or SeuratDisk. However, these tools rely on data type conversion, which can be computationally expensive. SCUBA implements a unified API for single-cell object formats to efficiently access data for exploratory analysis and visualization. It extracts from storage objects using FetchData methods a table with requested features, metadata, and reduction coordinates for each cell. The manuscript is clearly written, with a logical structure that effectively conveys the study's objective and organized in the following structure: 1) Introduction; 2) Methods including subsection Implementation and Operation; 3) Use cases; and 4) Conclusions. The “Use cases” section involve examples of Scuba usage: 1) application of FetchData function to retrieve features, metadata, and dimensionality reduction coordinates; 2) S3 generics and methods for similar tasks as in (1) including functions fetch_metadata and fetch_reduction; 3) speed tests of the functions for SeuratObjects stored in RDS format, SingleCellExperiment and Anndata in h5 formats;  3) presentation of example scripts for single-cell data exploration and visualization. While the issue of single-cell data interoperability is a well-recognized and pervasive challenge in the field, the manuscript in the current form does not appear to present a significant advancement or novel solution to address this problem comprehensively. It represents rather a set of functions that allows accessing data from various single-cell data objects needed for visualization purposes and should be a part of their scExploreR package rather than the standalone package.

 Other comments:

 1) The authors justify the need for SCUBA development by the fact that conversion between different data storage formats results in data loss upon conversion or this process is error-prone. Additionally, the rapid development of single-cell packages means that conversion functions are difficult to maintain and frequently break due to changes in other packages. I don’t understand why data is lost during conversion or why it is error-prone and how their approach overcome these limitations. For me, Scuba has exactly the same problems. In case of mentioned updates in single-cell third-party software and storage formats, Authors’ SCUBA code will require updates in the same way.  

 2) Authors should explain how different visualization software like CellxGene and many others retrieve features or cell metadata and benchmark them against SCUBA.

 3) One of the most common features of exploratory analysis is retrieval of feature values such as gene expression or chromatin accessibility from raw count or processed feature matrices. While this function is presented in Figure 2, it is omitted in the performance testing in Figure 4. Why? Can SCUBA extract, for example, gene expression values for a specified gene from huge matrices (500k cells) from SeuratObjects, SingleCellExperiments, Anndata objects in efficient manner? Authors show only retrieval of cell coordinates and cell metadata which are stored in smaller data structures.  

 4) It is not clear if Authors developed also a new faster version of FetchData for SeuratObject.

 5) The speed tests in Figure 4 are confusing. It’s not clear when Authors use native “FetchData” functions from Seurat/Scanpy and when from SCUBA. For example, Figure 4:  fetch_metadata is slower than “FetchData” from Scanpy for anndata?

 6) All the differences in run times in A-C are of the order of 0.5-2 seconds, so negligible for users analyzing the single-cell data.

 7) Authors state: “Analysis tools generally only accept one object class, forcing users to commit to a suite of packages at the beginning of an analysis. This restriction limits access to tools outside that suite, creating “walled gardens” that pose several challenges for single-cell analysis.” How Scuba overcomes this problem?

 8) The package lacks robust and well-organized documentation, such as that provided by the aforementioned zellkoverter, anndataR, or SeuratDisk. The use of the FetchData function is also unclear. The name exceptionally follows the Pascal case naming convention, unlike all other functions which follow the Camel case. If this is done deliberately, e.g., to highlight the use of the Seurat or SeuratObject packages, please consider including a namespace so that the user is aware of the external packages being employed. Alternatively, consider adding the fetch_data function to make the SCUBA package appear more like a closed, independent toolkit.

Are the conclusions about the tool and its performance adequately supported by the findings presented in the article?

No

Is the rationale for developing the new software tool clearly explained?

Partly

Is the description of the software tool technically sound?

Partly

Are sufficient details of the code, methods and analysis (if applicable) provided to allow replication of the software development and its use by others?

Partly

Is sufficient information provided to allow interpretation of the expected output datasets and any results generated using the tool?

Yes

Reviewer Expertise:

Single-cell Genomics, Bioinformatics

We confirm that we have read this submission and believe that we have an appropriate level of expertise to state that we do not consider it to be of an acceptable scientific standard, for reasons outlined above.

# Response

**Published:** 2025-05-27

**Authors:** William Showers

**Affiliations:** Division of Hematology, University of Colorado Anschutz Medical Campus, Aurora, Colorado, USA

Thank you for the time and effort invested in your review. We have modified the manuscript and package, and we feel it is stronger thanks to your feedback. Please see below for point-by-point responses.

*In this study, William M. Showers and colleagues developed SCUBA, an R package designed to facilitate the access to single-cell data stored in various formats commonly utilized by single-cell data analysis software in R (Seurat, Scran) or Python (Scanpy). These single-cell data storage formats include SeuratObject (Seurat), SingleCellExperiment (Scran, Bioconductor’s single-cell tools), Anndata (Scanpy). Interoperability between R-based and Python-based tools and data types is a real problem in bioinformatics. Several tools are typically used in this case, such as zellkonverter, anndataR, sceasy, or SeuratDisk. However, these tools rely on data type conversion, which can be computationally expensive. SCUBA implements a unified API for single-cell object formats to efficiently access data for exploratory analysis and visualization. It extracts from storage objects using FetchData methods a table with requested features, metadata, and reduction coordinates for each cell. The manuscript is clearly written, with a logical structure that effectively conveys the study's objective and organized in the following structure: 1) Introduction; 2) Methods including subsection Implementation and Operation; 3) Use cases; and 4) Conclusions. The “Use cases” section involve examples of Scuba usage: 1) application of FetchData function to retrieve features, metadata, and dimensionality reduction coordinates; 2) S3 generics and methods for similar tasks as in (1) including functions fetch_metadata and fetch_reduction; 3) speed tests of the functions for SeuratObjects stored in RDS format, SingleCellExperiment and Anndata in h5 formats;  3) presentation of example scripts for single-cell data exploration and visualization. While the issue of single-cell data interoperability is a well-recognized and pervasive challenge in the field, the manuscript in the current form does not appear to present a significant advancement or novel solution to address this problem comprehensively. It represents rather a set of functions that allows accessing data from various single-cell data objects needed for visualization purposes and should be a part of their scExploreR package rather than the standalone package.*

**Thank you for this assessment. The observation that SCUBA is a set of functions for accessing data from single-cell objects is correct, but we disagree that SCUBA should not be a standalone package. The functions provided by SCUBA are useful both in fetching data for interactive report generation (the usage context for scExploreR), and for static report generation by bioinformaticians. The consistent usage of SCUBA across object formats facilitates, for example, re-running a specific visualization script on data from a group that uses a different object class. SCUBA is a tool for developers to work seamlessly across different object classes, and we envision it as the basis of an analysis suite of static visualization packages, as well as the basis of scExploreR. We improved our documentation with examples of how users would integrate SCUBA into their analysis scripts.**

**Other comments:**

*1) The authors justify the need for SCUBA development by the fact that conversion between different data storage formats results in data loss upon conversion or this process is error-prone. Additionally, the rapid development of single-cell packages means that conversion functions are difficult to maintain and frequently break due to changes in other packages. I don’t understand why data is lost during conversion or why it is error-prone and how their approach overcome these limitations. For me, Scuba has exactly the same problems. In case of mentioned updates in single-cell third-party software and storage formats, Authors’ SCUBA code will require updates in the same way.*

**While it is true that SCUBA is vulnerable to changes in underlying object class implementation and must be maintained, we see this vulnerability as being less severe than with conversion packages. Conversion methods such as those mentioned aim to convert the entirety of each object class to another class, while SCUBA functions each extract small subsets of data. While still requiring active maintenance to track upstream changes, we believe this atomic approach is substantially less vulnerable to breaking changes that affect the entire package.**

*2) Authors should explain how different visualization software like CellxGene and many others retrieve features or cell metadata and benchmark them against SCUBA.*

**Thank you for the suggestion. We don’t feel this is in scope, as the API for data access in CELLxGENE and other visualization software is not exposed to end users in the way that Seurat’s FetchData is.**

*3) One of the most common features of exploratory analysis is retrieval of feature values such as gene expression or chromatin accessibility from raw count or processed feature matrices. While this function is presented in Figure 2, it is omitted in the performance testing in Figure 4. Why? Can SCUBA extract, for example, gene expression values for a specified gene from huge matrices (500k cells) from SeuratObjects, SingleCellExperiments, Anndata objects in efficient manner? Authors show only retrieval of cell coordinates and cell metadata which are stored in smaller data structures.*

**Thank you for pointing this out. The function mentioned for retrieval of gene expression data was benchmarked in figure 4A. The captions for figure 4C and figure 4A were switched, which incorrectly suggested that figure 4A was benchmarking fetch_reduction. We regret the confusion caused by this error and have corrected it in the revised version. Figure 4A has also been changed to more clearly reflect that the performance of the original Seruat FetchData is being compared to the methods added in SCUBA.**

*4) It is not clear if Authors developed also a new faster version of FetchData for SeuratObject.*

**We did not. Figure 4A has been updated to make this clear. In addition, we created a snake case fetch_data generic in response to comment 8, below. `fetch_data.Seurat` is simply a wrapper for Seurat’s FetchData, while `fetch_data.SingleCellExperiment` and `fetch_data.AnnDataR6` are added by SCUBA. This has been made clear in the manuscript and the updated documentation.**

*5) The speed tests in Figure 4 are confusing. It’s not clear when Authors use native “FetchData” functions from Seurat/Scanpy and when from SCUBA. For example, Figure 4:  fetch_metadata is slower than “FetchData” from Scanpy for anndata?*

**As mentioned above, we feel that the new generic fetch_data makes this clear. Figure 4B and figure 4C compare the `fetch_data` method in SCUBA to `fetch_metadata` and `fetch_reduction`, respectively.**

*6) All the differences in run times in A-C are of the order of 0.5-2 seconds, so negligible for users analyzing the single-cell data.*

**Thanks for this observation. We agree in the context of interactive analysis in an R session, but disagree in the context of interactive web apps that plot data from single-cell objects with many cells. Regardless, while this is a valid point, we feel the main benefit of SCUBA is the consistency in syntax across object formats.**

*7) Authors state: “Analysis tools generally only accept one object class, forcing users to commit to a suite of packages at the beginning of an analysis. This restriction limits access to tools outside that suite, creating “walled gardens” that pose several challenges for single-cell analysis.” How Scuba overcomes this problem?*

**While SCUBA does not fully overcome this problem, we see SCUBA as being a dependency of packages that would do so. We envision an analysis suite with packages for analyses such as differential gene expression and gene set enrichment analysis, as well as static and interactive visualizations. Packages built on SCUBA access functions will be flexible by default to input object class, and should be easier for end users to use compared to running a function from a conversion package, and then running an existing function from an analysis package. We also expect packages based on SCUBA to be easier to develop, since the access functions are easier to use than running class-specific access code, and have consistent syntax.**

*8) The package lacks robust and well-organized documentation, such as that provided by the aforementioned zellkoverter, anndataR, or SeuratDisk. The use of the FetchData function is also unclear. The name exceptionally follows the Pascal case naming convention, unlike all other functions which follow the Camel case. If this is done deliberately, e.g., to highlight the use of the Seurat or SeuratObject packages, please consider including a namespace so that the user is aware of the external packages being employed. Alternatively, consider adding the fetch_data function to make the SCUBA package appear more like a closed, independent toolkit.*

**We appreciate these observations. In addition to overhauling the function documentation for clarity, we added a pkgdown site at https://amc-heme.github.io/SCUBA/. In the site, we have added the “User Guide” vignette to the “Articles” tab, which contains a walkthrough of key functions in the SCUBA package for the access, exploration, and visualization of single-cell datasets.**

**As suggested, we created a new generic fetch_data, and moved the pascal case FetchData methods added by SCUBA to this generic. The fetch_data method for Seurat is a wrapper for Seurat’s FetchData method, and the fetch_data methods for SingleCellExperiment and anndata are added by SCUBA. In addition to improving clarity, the fetch_data generic removes the need for a dependency on Seurat.**

# Reviewer report: Reviewer response for version 1

**DOI:** 10.5256/f1000research.169727.r334007

**Published:** 2025-01-15

**Authors:** Kristian Ullrich

**Affiliations:** 1 Scientific IT group, Max Planck Institute for Evolutionary Biology, Plon, Germany

In this article the authors provide a valuable R package that implements a unified data access API for single-cell object formats like `anndata` objects (Python) and `Seurat/SingleCellExperiment` objects (R). The SCUBA API (implemented in R) keeps the original single-cell objects unmodified and use three data accession methods, namely `FetchData`, `fetch_metdata` and `fetch_reduction`. To more easily compare pre-processed data stored in common single-cell data formats, like `AnnData`, `SingleCellExperiment` (SCE) and `Seurat`, the authors fetch metadata from cells and genes from the original object and create a `data.frame` R object with the requested features. Using the `data.frame` object, downstream plotting function are provided to create plots in the `ggplot2` R package grammar.

**Code review**

 Given the dependency issues related to R-base version and Python version, I would encourage the authors to submit their package to either CRAN or Bioconductor R repository so that R package community standards are tested in a continious integration setup. The authors should try to alter their code to pass at least the R-CMD-check without warnings and errors.

 In the "Use cases" section, the authors claim that "Additional vignettes are provided on the SCUBA GitHub page", which is not true time of writing this review. Please add the mentioned vignettes to the GitHub page.

 The provided R functions should contain runnable examples. Building a vignette would help the community to present the basic functions from the R package and provide more background information, if needed. I would suggest the authors to include a github workflow to create the R package documentation, vignettes and convert it into a website e.g. with the `pkgdown` package or similar workflow.

**Software**

- I was able to install the software and run the examples given on the main github page, despite the need to pre-load the `Seurat` R package. As indicated in the Minor comments part, the three functions `reduction_names()`, `assay_names()` and `features_in_assay()` have been removed from the repository and the given example code block should be also removed from the github page.
- The Rmarkdown files on the github page https://github.com/amc-heme/SCUBA_Manuscript has been inspected and partially run. I have not re-created the subset data sets, however the code base seems to be fine.

**Major comments**

- If many genes are requested by the `FetchData` function, creating a single `data.frame` has the offsite to not benefit from sparse data structures leading to high memory footprints. Please implement some checks to prevent a user given the local resources to request too many gene features.
- Please remove "manually" from the sentence: "Currently, the most efective solution is to manually convert between object classes." Here, manually implies a programatically high efford to do so, which in my opinion is not true, given the existing converting tools like `SeuratWrapper`, `zellkonverter`, `SCEasy Converter` `scDIOR`. Please add the corresponding literature for the mentioned tools.
- In your R DESCRIPTION file you set the `reticulate` and `anndata` R packages as suggests, however for your API to be fully functional these packages needs to be imported. Please change accordingly.

**Minor comments**

- Please change the DESCRIPTION and add the corresponding author/creators in the github repository.
- Please alter the examples from your initial github page so that they work as expected. E.g. the `FetchData` function for Seurat objects is not pre-loaded, one need to first import Seurat `library(Seurat)`.
- Please remove the following example part from your github pages, sicne the functions have been removed and are not working anymore `reduction_names()`, `assay_names()` and `features_in_assay()`.
- Please update the corresponding `fetch_...` function main description and param field about what object types are supported, since the `AnnDataR6` class in most cases is supported but not mentioned.
- Please rephrase "Single-cell analysis can be inaccessible to bench scientists due to programming experience required, ...", in my opinion this sentence is formulated too harshly, as it is possible e.g. to convert and analyze single-cell objects with GUI-based solutions such as usegalaxy.eu and the SCEasy Converter plugin.
- Please provide a working example for a default user without admin priviliges how to setup and use `reticulate` in R for the usecase described here to access an `AnnData` object and how to install the prerequisite Python packages. The authors refer to the main `reticulate` documentation, however, showing a working code snippet example would be beneficial for scientists not fluent in both programming languages.
- Please remove "for all three object types" from Figure 5A "Common plotting script for all three object types", since the plotting function applies on the constructed intermediate `data.frame` object.
- Figure 6, please change the code example to use the R default pipe operator `|>` instead of the `magrittr` pipe operator `%>%` so that one would not need to pre-load the corresponding libraries.

Are the conclusions about the tool and its performance adequately supported by the findings presented in the article?

Partly

Is the rationale for developing the new software tool clearly explained?

Yes

Is the description of the software tool technically sound?

Partly

Are sufficient details of the code, methods and analysis (if applicable) provided to allow replication of the software development and its use by others?

Yes

Is sufficient information provided to allow interpretation of the expected output datasets and any results generated using the tool?

Yes

Reviewer Expertise:

Comparative Genomics, Bioinformatics, R Programming

I confirm that I have read this submission and believe that I have an appropriate level of expertise to confirm that it is of an acceptable scientific standard, however I have significant reservations, as outlined above.

## References

[1] : Comparison of visualization tools for single-cell RNAseq data. *NAR Genom Bioinform* .2020;2(3) : 10.1093/nargab/lqaa052 lqaa052 10.1093/nargab/lqaa052 32766548 PMC7391988

[2] : scDIOR: single cell RNA-seq data IO software. *BMC Bioinformatics* .2022;23(1) : 10.1186/s12859-021-04528-3 16 10.1186/s12859-021-04528-3 34991457 PMC8734364

# Response

**Published:** 2025-05-23

**Authors:** William Showers

**Affiliations:** Division of Hematology, University of Colorado Anschutz Medical Campus, Aurora, Colorado, USA

Thank you for the time and effort invested in your review. We have modified the manuscript and package, and we feel it is stronger thanks to your feedback. Please see below for point-by-point responses.

*In this article the authors provide a valuable R package that implements a unified data access API for single-cell object formats like `anndata` objects (Python) and `Seurat/SingleCellExperiment` objects (R). The SCUBA API (implemented in R) keeps the original single-cell objects unmodified and use three data accession methods, namely `FetchData`, `fetch_metdata` and `fetch_reduction`. To more easily compare pre-processed data stored in common single-cell data formats, like `AnnData`, `SingleCellExperiment` (SCE) and `Seurat`, the authors fetch metadata from cells and genes from the original object and create a `data.frame` R object with the requested features. Using the `data.frame` object, downstream plotting function are provided to create plots in the `ggplot2` R package grammar.*

***Code review***

*Given the dependency issues related to R-base version and Python version, I would encourage the authors to submit their package to either CRAN or Bioconductor R repository so that R package community standards are tested in a continious integration setup. The authors should try to alter their code to pass at least the R-CMD-check without warnings and errors.*

**Thank you for this valuable feedback. While we do not intend to submit this package to CRAN or Bioconductor, we recognize the value of continuous integration and have ensured that the code now passes the R-CMD-check without warnings or errors.**

*In the "Use cases" section, the authors claim that "Additional vignettes are provided on the SCUBA GitHub page", which is not true time of writing this review. Please add the mentioned vignettes to the GitHub page.*

**We have added a vignette with examples of all major SCUBA functions (see the comment below).**

*The provided R functions should contain runnable examples. Building a vignette would help the community to present the basic functions from the R package and provide more background information, if needed. I would suggest the authors to include a github workflow to create the R package documentation, vignettes and convert it into a website e.g. with the `pkgdown` package or similar workflow.*

**We appreciate this feedback, and have improved our documentation to address these important observations. Specifically, we have added a pkgdown site at https://amc-heme.github.io/SCUBA/. Here, we have added the “User Guide” vignette to the “Articles” tab, which contains a walkthrough of key functions in the SCUBA package for the access, exploration, and visualization of single-cell datasets. The vignette shows usage examples for all three object types currently supported by SCUBA. In addition to adding the vignette, we updated the function documentation to improve clarity and provide usage examples.**

***Software***

*I was able to install the software and run the examples given on the main github page, despite the need to pre-load the `Seurat` R package. As indicated in the Minor comments part, the three functions `reduction_names()`, `assay_names()` and `features_in_assay()` have been removed from the repository and the given example code block should be also removed from the github page.*

**Thanks for letting us know about this. We have removed reduction_names() and assay_names() from the documentation to avoid this confusion. Features_in_assay() was added back to the package, and documentation on usage of this function is now on our pkgdown site, in the “User Guide” article.**

*The Rmarkdown files on the github page https://github.com/amc-heme/SCUBA_Manuscript have been inspected and partially run. I have not re-created the subset data sets, however the code base seems to be fine.*

**We have double-checked the code in amc-heme/SCUBA_Manuscript internally for consistent outputs. Please let us know if you notice further issues with the code in the manuscript repo.**

***Major comments***

*If many genes are requested by the `FetchData` function, creating a single `data.frame` has the offsite to not benefit from sparse data structures leading to high memory footprints. Please implement some checks to prevent a user given the local resources to request too many gene features.*

**Thank you for this observation. We don’t feel that it is necessary to prevent users from doing this, but we have added a warning to users (shown below) in this scenario. The warning appears when 1000 or more genes are requested.**

**"A very large number of features was requested (<> features). fetch_data is not intended to be used with feature queries of this length. Data is returned in a dense format, so the memory usage of the output may be very large. Also, this query may take a while to complete."**

*Please remove "manually" from the sentence: "Currently, the most efective solution is to manually convert between object classes." Here, manually implies a programatically high efford to do so, which in my opinion is not true, given the existing converting tools like `SeuratWrapper`, `zellkonverter`, `SCEasy Converter` `scDIOR`. Please add the corresponding literature for the mentioned tools.*

**Thank you for pointing this out. The sentence was changed as follows in the updated manuscript:**

**"All major single-cell analysis packages implement conversion functions, and third-party packages such as sceasy, Zellconverter, and scDIOR are specifically designed for these conversions. Additionally, SeruatWrapper implements conversion functions that allow several otherwise-incompatible single-cell analysis packages to be used on Seurat objects."**

**Citations for the corresponding literature have also been added to the revised manuscript.**

*In your R DESCRIPTION file you set the `reticulate` and `anndata` R packages as suggests, however for your API to be fully functional these packages needs to be imported. Please change accordingly.*

**We appreciate this observation. While we prefer not to require users to install anndata and reticulate if they are not using anndata objects, we have added a conditional statement in all exported AnnDataR6 methods to check if these packages are installed, and throw an error directing users to install these packages if they are not.**

***Minor comments***

*Please change the DESCRIPTION and add the corresponding author/creators in the github repository.*

**This change was made.**

*Please alter the examples from your initial github page so that they work as expected. E.g. the `FetchData` function for Seurat objects is not pre-loaded, one need to first import Seurat `library(Seurat)`.*

**To keep users from having to import Seurat to use our FetchData methods, we moved them to a new generic defined within our package (fetch_data).**

*Please remove the following example part from your github pages, since the functions have been removed and are not working anymore `reduction_names()`, `assay_names()` and `features_in_assay()`.*

**All three functions have been removed from the README on our GitHub page. We added `features_in_assay()` back to SCUBA, and have added documentation for the function in our user guide vignette.**

*Please update the corresponding `fetch_...` function main description and param field about what object types are supported, since the `AnnDataR6` class in most cases is supported but not mentioned.*

This has been corrected. All fetch_* functions and all functions that take an object as a parameter now mention all object types corrected. The description text for the `object` parameter is now also consistent across all functions.

*Please rephrase "Single-cell analysis can be inaccessible to bench scientists due to programming experience required, ...", in my opinion this sentence is formulated too harshly, as it is possible e.g. to convert and analyze single-cell objects with GUI-based solutions such as usegalaxy.eu and the SCEasy Converter plugin.*

**We have modified this sentence to the following: “Single-cell analysis requires programming experience for full customization of analysis and visualization, and different object formats make it even more difficult for biologists to analyze data”.**

*Please provide a working example for a default user without admin privileges how to setup and use `reticulate` in R for the usecase described here to access an `AnnData` object and how to install the prerequisite Python packages. The authors refer to the main `reticulate` documentation, however, showing a working code snippet example would be beneficial for scientists not fluent in both programming languages.*

**Thanks for this suggestion. We have added this to the README, under the section “Additional Installation for anndata Objects”.**

*Please remove "for all three object types" from Figure 5A "Common plotting script for all three object types", since the plotting function applies on the constructed intermediate `data.frame` object.*

**This has been changed. The sentence now reads “Example script for visualizing expression of a gene by cluster in a density plot.”**

*Figure 6, please change the code example to use the R default pipe operator `|>` instead of the `magrittr` pipe operator `%>%` so that one would not need to pre-load the corresponding libraries.*

**This change has been made. Please see figure 6 in the revised submission.**

# Reviewer report: Reviewer response for version 1

**DOI:** 10.5256/f1000research.169727.r346283

**Published:** 2024-12-17

**Authors:** Huamei Li

**Affiliations:** 1 Nanjing University, Nanjing, Jiangsu, China

Showers et al. developed the SCUBA R package as a unified API for single-cell data analysis. In single-cell and spatial transcriptomics studies, data format conversion between Seurat, SingleCellExperiment, and AnnData often presents challenges, leading to obstacles in downstream analyses. While several tools have been developed to address data format conversion issues, SCUBA provides a unified and user-friendly interface for handling multiple formats and simplifies single-cell data processing workflows, making it a promising tool for practical applications. However, several concerns regarding SCUBA require further clarification:

 1) The README file available at https://github.com/amc-heme/SCUBA/tree/main does not include examples demonstrating how SCUBA reads Seurat, SingleCellExperiment, and AnnData formats from local files. Additionally, it is unclear whether SCUBA addresses compatibility issues arising from different versions of these formats, such as Seurat 4.0+ versus Seurat 5.0+. Can SCUBA facilitate seamless data format conversion and overcome challenges posed by version discrepancies?

 2) While SCUBA provides application examples for single-cell datasets, it remains unclear whether the tool can be effectively extended to spatial transcriptomics datasets generated by different platforms. Clarification on its applicability to spatial datasets would be beneficial.

 3) To enhance visualization capabilities, it is recommended that SCUBA include optional smoothing methods for displaying the expression distribution of feature genes. This would allow for better identification of regions with concentrated expression of specific features.

 4) The SCUBA documentation could be further improved by providing more detailed explanations of the functions, their usage, and intended purposes. Additionally, including a comprehensive analysis example that integrates both single-cell and spatial transcriptomics data would help illustrate the full workflow and practical utility of SCUBA.

Are the conclusions about the tool and its performance adequately supported by the findings presented in the article?

Yes

Is the rationale for developing the new software tool clearly explained?

Yes

Is the description of the software tool technically sound?

Yes

Are sufficient details of the code, methods and analysis (if applicable) provided to allow replication of the software development and its use by others?

Partly

Is sufficient information provided to allow interpretation of the expected output datasets and any results generated using the tool?

Yes

Reviewer Expertise:

Bioinformatics, Immunogenetics, Single-cell and spatial technologies

I confirm that I have read this submission and believe that I have an appropriate level of expertise to confirm that it is of an acceptable scientific standard.

# Response

**Published:** 2025-05-23

**Authors:** William Showers

**Affiliations:** Division of Hematology, University of Colorado Anschutz Medical Campus, Aurora, Colorado, USA

Thank you for the time and effort invested in your review. We have modified the manuscript and package, and we feel it is stronger thanks to your feedback. Please see below for point-by-point responses.

*Showers et al. developed the SCUBA R package as a unified API for single-cell data analysis. In single-cell and spatial transcriptomics studies, data format conversion between Seurat, SingleCellExperiment, and AnnData often presents challenges, leading to obstacles in downstream analyses. While several tools have been developed to address data format conversion issues, SCUBA provides a unified and user-friendly interface for handling multiple formats and simplifies single-cell data processing workflows, making it a promising tool for practical applications. However, several concerns regarding SCUBA require further clarification:*

*1) The README file available at https://github.com/amc-heme/SCUBA/tree/main does not include examples demonstrating how SCUBA reads Seurat, SingleCellExperiment, and AnnData formats from local files. Additionally, it is unclear whether SCUBA addresses compatibility issues arising from different versions of these formats, such as Seurat 4.0+ versus Seurat 5.0+. Can SCUBA facilitate seamless data format conversion and overcome challenges posed by version discrepancies?*

**Thank you for this observation. We have added new documentation via a pkgdown website ( https://amc-heme.github.io/SCUBA/). The Article “User Guide” contains a note on how each object class is loaded. SCUBA is seamlessly compatible with Seurat v4 and v5 objects. We have added a note on this to the README.**

*2) While SCUBA provides application examples for single-cell datasets, it remains unclear whether the tool can be effectively extended to spatial transcriptomics datasets generated by different platforms. Clarification on its applicability to spatial datasets would be beneficial.*

**SCUBA partially supports spatial datasets. Aspects of spatial datasets that can be expressed as a counts matrix can be loaded into SCUBA, but we do not yet support the loading of image files. We have added a note on this to the README.**

*3) To enhance visualization capabilities, it is recommended that SCUBA include optional smoothing methods for displaying the expression distribution of feature genes. This would allow for better identification of regions with concentrated expression of specific features.*

**Thank you for the suggestion. While we agree that this is a valuable approach, we feel this is out of scope for the current tool, and welcome users to apply their own smoothing methods in plotting scripts based on SCUBA.**

*4) The SCUBA documentation could be further improved by providing more detailed explanations of the functions, their usage, and intended purposes. Additionally, including a comprehensive analysis example that integrates both single-cell and spatial transcriptomics data would help illustrate the full workflow and practical utility of SCUBA.*

**Thank you for the suggestion. We have added the suggested documentation to the README and the user guide vignette on the pkgdown website. We have also updated function documentation in SCUBA v.1.1.0.**
